# Carfilzomib–lenalidomide–dexamethasone vs lenalidomide–dexamethasone in relapsed multiple myeloma by previous treatment

**DOI:** 10.1038/bcj.2017.31

**Published:** 2017-04-21

**Authors:** M A Dimopoulos, A K Stewart, T Masszi, I Špička, A Oriol, R Hájek, L Rosiñol, D Siegel, G G Mihaylov, V Goranova-Marinova, P Rajnics, A Suvorov, R Niesvizky, A Jakubowiak, J San-Miguel, H Ludwig, S Ro, S Aggarwal, P Moreau, A Palumbo

**Affiliations:** 1 School of Medicine, National and Kapodistrian University of Athens, Athens, Greece; 2 Division of Hematology–Oncology, Mayo Clinic, Scottsdale, AZ, USA; 3 Department of Hematology and Stem-Cell Transplantation, St. István and St. László Hospital, Semmelweis University, Budapest, Hungary; 4 Department of Internal Medicine, Charles University in Prague, First Faculty of Medicine and General Teaching Hospital, Prague, Czech Republic; 5 Department of Clinical Hematology, Institut Català d'Oncologia–Hospital Universitari Germans Trias i Pujol, Institut Josep Carreras, Barcelona, Spain; 6 Faculty of Medicine, University Hospital Ostrava, Faculty of Medicine, University of Ostrava, Ostrava, Czech Republic; 7 Department of Hematology, Hospital Clínic de Barcelona, Barcelona, Spain; 8 Division of Multiple Myeloma, John Theurer Cancer Center at Hackensack University Medical Center, Hackensack, NJ, USA; 9 Hematological Clinic, Queen Joanna University Hospital, Sofia, Bulgaria; 10 Hematology Clinic, University Multiprofile Hospital for Active Treatment ‘Sv. Georgi' and Medical University, Plovdiv, Bulgaria; 11 Department of Hematology, Mór Kaposi Teaching Hospital, Kaposvár, Hungary; 12 First Republican Clinical Hospital of Udmurtia, Izhevsk, Russia; 13 Multiple Myeloma Center, Weill Cornell Medical College, New York, NY, USA; 14 Myeloma Program, University of Chicago Medicine, Chicago, IL, USA; 15 Clinical and Translational Medicine, Clinica Universidad de Navarra–Centro de Investigación Médica Aplicada, IDISNA, CIBERONC, Pamplona, Spain; 16 Department of Medicine, Wilhelminen Cancer Research Institute, Wilhelminenspital, Vienna, Austria; 17 Onyx Pharmaceuticals, Inc., An Amgen Subsidiary, South San Francisco, CA, USA; 18 Department of Hematology, University of Nantes, Nantes, France; 19 Myeloma Unit, Department of Oncology, University of Turin, Turin, Italy

## Abstract

Carfilzomib, a proteasome inhibitor, is approved as monotherapy and in combination with dexamethasone or lenalidomide–dexamethasone (Rd) for relapsed or refractory multiple myeloma. The approval of carfilzomib–lenalidomide–dexamethasone (KRd) was based on results from the randomized, phase 3 study ASPIRE (NCT01080391), which showed KRd significantly improved progression-free survival (PFS) vs Rd (median 26.3 vs 17.6 months; hazard ratio (HR)=0.690; *P*=0.0001). This subgroup analysis of ASPIRE evaluated KRd vs Rd by number of previous lines of therapy and previous exposure to bortezomib, thalidomide or lenalidomide. Treatment with KRd led to a 12-month improvement in median PFS vs Rd after first relapse (HR 0.713) and a 9-month improvement after ⩾2 previous lines of therapy (HR 0.720). Treatment with KRd led to an approximate 8-month improvement vs Rd in median PFS in bortezomib-exposed patients (HR 0.699), a 15-month improvement in thalidomide-exposed patients (HR 0.587) and a 5-month improvement in lenalidomide-exposed patients (HR 0.796). Objective response and complete response or better rates were higher with KRd vs Rd, irrespective of previous treatment. KRd had a favorable benefit–risk profile and should be considered an appropriate treatment option for patients with 1 or ⩾2 previous lines of therapy and those previously exposed to bortezomib, thalidomide or lenalidomide.

## Introduction

Important patient- and disease-specific factors, including previous treatments, are considered when selecting a second- or third-line therapy for patients with relapsed multiple myeloma (MM).^1, 2^ In the United States, bortezomib- and lenalidomide-based regimens are the most commonly used first- and second-line therapies for patients with MM;^3^ these regimens also are endorsed by international clinical guidelines.^2, 4^

As patients progress through multiple lines of bortezomib- and lenalidomide-based regimens in early lines of therapy, they may develop treatment-resistant disease. One study in patients with relapsed MM reported a median duration of response of approximately 10 months in patients with one prior regimen and a median duration of response of approximately 6 months in patients with three prior regimens.^5^ Given the prognostic value of the number of previous lines of therapy and the frequent use of bortezomib and lenalidomide as front-line therapy, there is a critical need to evaluate the clinical effect of previous treatment on the efficacy and safety of novel treatment strategies used for patients with relapsed MM.

Carfilzomib is a selective and irreversible epoxyketone proteasome inhibitor that is approved in the United States for use in combination with dexamethasone or lenalidomide plus dexamethasone for the treatment of patients with relapsed or refractory MM (1–3 previous lines of therapy) and as a single agent for the treatment of patients with relapsed or refractory MM (1 or more previous lines of therapy); the agent has also been approved in Europe for use in combination with lenalidomide and dexamethasone (Rd) for the treatment of patients with MM who have received at least one previous line of therapy.^6^ The approvals for the combination of carfilzomib, lenalidomide and dexamethasone (KRd) were based on results from a preplanned interim analysis of the randomized, phase 3 ASPIRE trial, which evaluated KRd vs Rd in patients with relapsed MM (1–3 previous lines of therapy). Treatment with KRd led to a significant reduction in the risk of disease progression or death when compared with Rd (hazard ratio (HR) 0.69; 95% confidence interval (95% CI): (0.57, 0.83); *P*=0.0001), demonstrating the superiority of KRd vs Rd for the primary endpoint of progression-free survival (PFS). The overall response rate (ORR) was higher in the KRd group vs the Rd group (87.1% (95% CI: 83.4, 90.3) vs 66.7% (95% CI: 61.8, 71.3); odds ratio, 3.47 (95% CI: 2.41, 5.00)), including a complete response (CR) or better in 31.8% and 9.3% of patients in the two groups, respectively. Patients in the KRd group consistently reported superior health-related quality of life relative to those in the Rd group during 18 cycles of treatment.^7^

As the treatment of patients with relapsed and/or refractory MM who have received multiple lines of therapy is challenging, we performed a subgroup analysis of the interim results from the ASPIRE study to evaluate the efficacy and safety of KRd vs Rd by the number of previous lines of therapy (1 vs ⩾2), previous exposure to bortezomib, thalidomide or lenalidomide, and refractory status to bortezomib, thalidomide or lenalidomide in any previous regimen.

## Materials and methods

### Study design and patients

ASPIRE was a randomized, open-label, phase 3 study that evaluated KRd vs Rd in adults with relapsed MM (1–3 previous lines of therapy). Patients previously treated with bortezomib were eligible if they did not experience disease progression during therapy; patients previously treated with lenalidomide were eligible provided they did not discontinue treatment owing to adverse effects, have disease progression during the first 3 months of therapy with Rd, or have progression at any time during treatment if Rd was their most recent treatment. All patients provided written informed consent.

The primary endpoint was PFS in the intent-to-treat (ITT) population. Secondary endpoints included overall survival, ORR, health-related quality of life and safety. Here we present the subgroup analyses of PFS and ORR. Overall survival was not included in this subgroup analysis, as the data were not mature and were awaiting final analysis. The subgroup analysis for PFS and ORR by prior lines, prior exposure and disease resistance were preplanned; however, results are for hypothesis-generating purposes are not adjusted for multiplicity. Thus, they should be interpreted descriptively. The study protocol was approved by the institutional review boards or ethics committees of all participating sites and was conducted in accordance with the International Conference on Harmonisation Guidelines for Good Clinical Practice and the Declaration of Helsinki. ASPIRE is registered with ClinicalTrials.gov (NCT01080391). Full details regarding the ASPIRE study design have been published.^7^

### Assessments

Disease progression and treatment response were assessed centrally in blinded manner by an independent review committee in accordance with International Myeloma Working Group Uniform Response Criteria.^8^ Minimal response was defined according to European Group for Blood and Marrow Transplant criteria.^9, 10^

The incidence and severity of adverse events (AEs) were recorded until 30 days after the last dose of study treatment. AEs were graded according to the National Cancer Institute Common Terminology Criteria for Adverse Events (v4.0).^11^

### Statistical analysis

Efficacy analyses were based on the ITT population. The safety population included all patients who received ⩾1 dose of study treatment. Subgroups were defined based on dichotomous baseline variables: previous lines of therapy (1 vs ⩾2), previous treatment with bortezomib (yes vs no), previous treatment with thalidomide (yes vs no), previous treatment with lenalidomide (yes vs no), refractory status to any previous bortezomib treatment (yes vs no), refractory status to thalidomide (yes vs no) in any previous regimen and refractory status to lenalidomide any previous regimen (yes vs no). Reported *P*-values for this subgroup analysis are one-sided, unadjusted for multiple comparisons and are descriptive in nature.

## Results

### Patient disposition

In total, 792 patients were enrolled and randomized to treatment with KRd (*n*=396) or Rd (*n*=396), as previously reported.^7^ A total of 341 patients had received one prior line of therapy (KRd, *n*=184; Rd, *n*=157), and 451 patients had received two or more prior lines of therapy (KRd, *n*=212; Rd, *n*=239). Patients with 1 or ⩾2 previous lines of therapy generally had similar baseline characteristics; within these subgroups, baseline characteristics were also generally well-balanced between the treatment arms (Table 1). However, baseline demographics and disease characteristics may not be relevant at the time of relapse. Median duration of treatment among patients who had received one previous line of therapy was 83.0 weeks in the KRd group and 64.0 weeks in the Rd group; among patients who had received ⩾2 previous lines of therapy, median duration of treatment was 90.6 weeks in the KRd group and 52.0 weeks in the Rd group. Other important demographic and clinical characteristics are summarized by previous lines of therapy (1 vs ⩾2) and treatment group (KRd vs Rd) in Table 1.

In all, 521 patients (65.8%) had received previous bortezomib (KRd, *n*=261; Rd, *n*=260); 118 (14.9%) had disease refractory to bortezomib in any previous regimen (KRd, *n*=60; Rd, *n*=58). In total, 347 (43.8%) had received previous thalidomide (KRd, *n*=176, Rd, *n*=171); 126 (15.9%) were refractory to thalidomide in any previous regimen (KRd, *n*=61; Rd, *n*=65) and 157 patients (19.8%) had received previous lenalidomide (KRd, *n*=79; Rd, *n*=78). A total of 57 patients (7.2%) were refractory to lenalidomide in any previous regimen, 26 (3.3%) were refractory to bortezomib and lenalidomide in any previous regimen and 33 (4.2%) were refractory to bortezomib and thalidomide in any previous regimen.

The cutoff date for the interim analysis was 16 June 2014.^7^

### Efficacy by line of therapy

**Figure 1:**
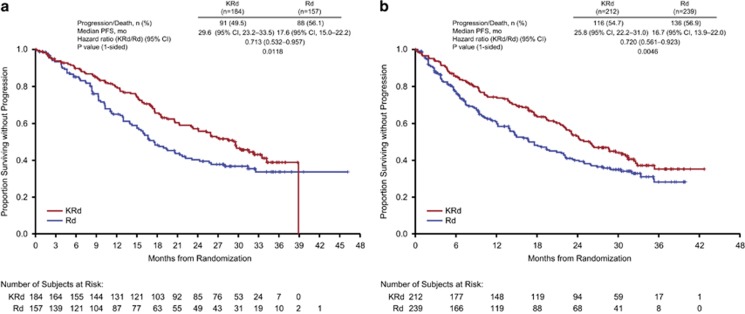
Kaplan–Meier estimate of PFS in patients with (**a**) 1 previous line of therapy and (**b**) ⩾2 previous lines of therapy.

Consistent with the overall results for the ITT population, the addition of carfilzomib to Rd reduced the risk of disease progression or death in patients who had received 1 previous line of therapy or ⩾2 previous lines of therapy (Figures 1a and b). Among patients with one previous line of therapy, the median PFS was 29.6 months in the KRd group and 17.6 months in the Rd group (HR=0.713; 95% CI: 0.532, 0.957; *P*=0.0118). Among patients with ⩾2 previous lines of therapy, the median PFS was 25.8 months in the KRd group and 16.7 months in the Rd group (HR=0.720; 95% CI: 0.561, 0.923; *P*=0.0046).

Response rates by line of therapy are shown in Table 2. Consistent with the overall results for the ITT population,^7^ among patients who received one previous line of therapy, the ORR was higher in the KRd group compared with the Rd group (87.0% vs 70.1%), with an odds ratio of 2.85 (95% CI: 1.65, 4.93). Similarly, among patients who received ⩾2 previous lines of therapy, the ORR was higher in the KRd group vs the Rd group (87.3% vs 64.4%), with an odds ratio of 3.78 (95% CI: 2.33, 6.13). Furthermore, regardless of the number of previous lines of therapy, a larger proportion of patients in the KRd achieved a CR or better than those in the Rd group (1 previous line: 33.7% vs 7.0% ⩾2 previous lines: 30.2% vs 10.9%).

### Efficacy by type of previous treatment

**Figure 2:**
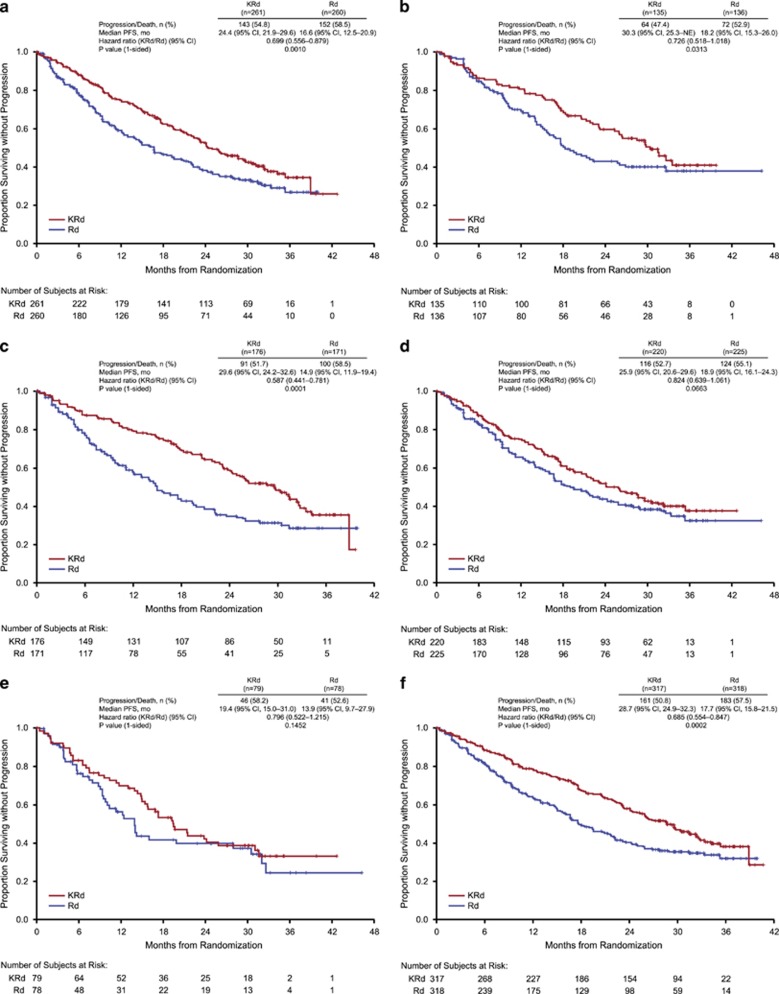
Kaplan–Meier estimate of PFS in patients with (**a**) previous bortezomib exposure, (**b**) no previous bortezomib exposure, (**c**) previous thalidomide exposure, (**d**) no previous thalidomide exposure, (**e**) previous lenalidomide exposure and (**f**) no previous lenalidomide exposure.

Also consistent with the overall results for the ITT population, treatment with KRd resulted in longer PFS compared with Rd, irrespective of previous exposure to bortezomib, thalidomide or lenalidomide (Figures 2a–f). The median PFS for KRd vs Rd was 24.4 vs 16.6 months for patients with previous bortezomib exposure (*n*=521; HR=0.699; 95% CI: 0.556, 0.879; *P*=0.0010) and 30.3 vs 18.2 months for patients without previous bortezomib exposure (*n*=271; HR=0.726, 95% CI: 0.518, 1.018; *P*=0.0313). The median PFS for KRd vs Rd was 29.6 vs 14.9 months for patients with previous thalidomide exposure (*n*=347; HR=0.587, 95% CI: 0.441, 0.781; *P*=0.0001), and 25.9 vs 18.9 months for patients without previous thalidomide exposure (*n*=445; HR=0.824, 95% CI: 0.639, 1.061; *P*=0.0663). The median PFS for KRd vs Rd was 19.4 vs 13.9 months for patients with previous lenalidomide exposure (*n*=157; HR=0.796; 95% CI: 0.522, 1.215; *P*=0.1452) and 28.7 vs 17.7 months for patients without previous lenalidomide exposure (*n*=635; HR=0.685; 95% CI: 0.554, 0.847; *P*=0.0002).

A consistency of benefit for KRd vs Rd in terms of PFS was also observed in patients with disease refractory to bortezomib, thalidomide or lenalidomide in any previous regimen. The median PFS for KRd vs Rd was 22.3 vs 19.4 months for patients with disease refractory to bortezomib (HR=0.799; 95% CI: 0.492, 1.297; *P*=0.1810), 24.1 vs 13.0 months for patients refractory to thalidomide (HR=0.599; 95% CI: 0.390, 0.918; *P*=0.0089), and 11.3 vs 9.0 months for patients refractory to lenalidomide (HR=0.571; 95% CI: 0.283, 1.149; *P*=0.0555).

Response by previous treatment is shown in Table 3. The ORRs were 86.2% (KRd) vs 63.5% (Rd) in patients with previous bortezomib exposure (odds ratio, 3.60 (95% CI: 2.33, 5.55)) and 88.9% (KRd) vs 72.8% (Rd) in patients without previous bortezomib exposure (odds ratio, 2.99 (95% CI: 1.55, 5.76)). In patients with previous thalidomide exposure, the ORRs were 88.6% (KRd) and 60.8% (odds ratio, 5.03 (95% CI: 2.88, 8.78)); in patients without previous thalidomide exposure, the ORRs were 85.9% vs 71.1% for KRd vs Rd (odds ratio, 2.48 (95% CI: 1.54, 3.99)). The ORRs were 81.0% (KRd) vs 50.0% (Rd) in patients with previous lenalidomide exposure (odds ratio, 4.27 (95% CI: 2.08, 8.73)) and 88.6% (KRd) vs 70.8% (Rd) in patients without previous lenalidomide exposure (odds ratio, 3.23 (95% CI: 2.11, 4.92)). The rates of CR or better were 29.9% (KRd) vs 9.6% (Rd) in patients with previous bortezomib exposure and 35.6% (KRd) vs 8.8% (Rd) in patients without previous bortezomib exposure. The rates of CR or better were 34.1% (KRd) vs 9.4% (Rd) in patients with previous thalidomide exposure and 30.0% (KRd) vs 9.3% (Rd) in patients without previous thalidomide exposure. In patients with previous lenalidomide exposure, the rates of CR or better were 24.1% (KRd) vs 5.1% (Rd); in patients without previous lenalidomide exposure, the rates of CR or better were 33.8% (KRd) vs 10.4% (Rd). For patients refractory to bortezomib, thalidomide or lenalidomide, the ORRs for KRd vs Rd were 80.0% vs 60.3% (odds ratio, 2.63; 95% CI, 1.16–5.99), 86.9% vs 52.3% (odds ratio, 6.04; 95% CI, 2.48–14.69) and 69.0% vs 25.0% (odds ratio, 6.67; 95% CI, 2.09–21.31), respectively.

### Safety by lines of therapy

In the subgroups that received 1 and ⩾2 previous lines of therapy, 336 (KRd, *n*=182; Rd, *n*=154) and 445 patients (KRd, *n*=210; Rd, *n*=235), respectively, received ⩾1 dose of study drug and were evaluable for safety. Any grade AEs occurred in 97.8% (KRd) and 99.4% (Rd) of patients who received 1 previous line of therapy and in 96.2% (KRd) and 95.7% (Rd) of patients who received ⩾2 previous lines of therapy. Common AEs and AEs of interest of any grade by line of therapy are shown in Table 4. Notable AEs included hypertension (preferred term; 1 previous line: 11.5% (KRd) vs 4.5% (Rd); ⩾2 previous lines: 16.7% (KRd) vs 8.5% (Rd)), acute renal failure (grouped term; 1 previous line: 11.0% (KRd) vs 6.5% (Rd); ⩾2 previous lines: 6.2% (KRd) vs 7.7% (Rd)) and dyspnea (preferred term; 1 previous line: 23.1% (KRd) vs 14.3% (Rd); ⩾2 previous lines: 16.2% (KRd) vs 15.3% (Rd)).

Common AEs of grade ⩾3 by line of therapy are shown in Table 5. AEs of grade ⩾3 occurred in 85.7% (KRd) and 79.9% (Rd) of patients who received 1 previous line of therapy and in 81.9% (KRd) and 81.3% (Rd) of patients who received ⩾2 previous lines of therapy. No AEs of grade ⩾3 occurred ⩾5% more frequently with KRd vs Rd in patients who received one previous line of therapy. Hypokalemia was the only AE of grade ⩾3 that occurred ⩾5% more frequently with KRd (11.0%) vs Rd (3.4%) in patients who received ⩾2 previous lines of therapy. In the KRd group, neutropenia was the only grade ⩾3 AE that occurred ⩾5% more frequently after ⩾2 previous lines of therapy (32.4%) vs 1 previous line of therapy (26.4%).

Serious AEs were observed in 63.2% (KRd) and 53.9% (Rd) of patients who received 1 previous line of therapy and 56.7% (KRd) and 53.6% (Rd) of patients who received ⩾2 previous lines of therapy. In total, 28.6% of KRd patients and 22.7% of Rd patients who had received 1 previous line of therapy discontinued any study drug owing to an AE; 23.8% of KRd patients and 26.4% of Rd patients who had received ⩾2 previous lines of therapy discontinued any study drug for this reason. Nine KRd patients (4.9%) and nine Rd patients (5.8%) who had received one previous line of therapy died while receiving study treatment or within 30 days of the last dose of study drug; 21 KRd patients (10.0%) and 24 Rd patients (10.2%) who had received ⩾2 previous lines of therapy died while receiving study treatment.

Among patients who received one previous line of therapy, AEs leading to permanent discontinuation of study treatment in two or more patients in any subgroup were thrombocytopenia (KRd, *n*=0; Rd, *n*=2), myocardial infarction (KRd, *n*=2 (both grade 5); Rd, *n*=0), acute myocardial infarction (KRd, *n*=2; Rd, *n*=2), death (KRd, *n*=2; Rd, *n*=1), fatigue (KRd, *n*=0; Rd, *n*=2), pneumonia (KRd, *n*=3; Rd, *n*=0), acute myeloid leukemia (KRd, *n*=2 (1 grade 5); Rd, *n*=0), colon cancer (KRd, *n*=2; Rd, *n*=0) and rash (KRd, *n*=0; Rd, *n*=2). Among patients who had received ⩾2 previous lines of therapy, AEs leading to permanent discontinuation of study treatment in two or more patients in any subgroup were cardiac failure (KRd, *n*=1 (grade 5); Rd, *n*=2 (both grade 5)), septic shock (KRd, *n*=2 (1 grade 5 event); Rd, *n*=1 (grade 5)), pneumonia (KRd, *n*=1 (grade 5); Rd, *n*=3 (2 grade 5 events)), acute myeloid leukemia (KRd, *n*=0; Rd, *n*=2 (1 grade 5 event)), myelodysplastic syndrome (KRd, *n*=0; Rd, *n*=2 (both grade 5)), cerebrovascular accident (KRd, *n*=0; Rd, *n*=2), acute renal failure (KRd, *n*=0; Rd, *n*=2 (1 grade 5 event)) and pulmonary embolism (KRd, *n*=0; Rd, *n*=2 (1 grade 5 event)).

## Discussion

This subgroup analysis of the ASPIRE study indicates that KRd improved outcomes in patients in their first relapse (1 previous line of therapy), patients with ⩾2 previous lines of therapy, patients with or without previous exposure to bortezomib, thalidomide or lenalidomide, and in patients refractory to bortezomib or refractory to thalidomide. Given the challenges of treatment in the relapsed and/or refractory setting, where the efficacy of anti-MM agents often declines with successive lines of therapy,^5^ it is an important finding that the HRs for PFS consistently favored KRd across all the subgroups based on previous treatment history. Treatment with KRd led to a 12-month improvement in median PFS vs Rd after first relapse and a 9-month improvement in median PFS vs Rd in patients with ⩾2 previous lines of therapy, with comparable HRs. Similarly, treatment with KRd led to an approximate 8-month improvement vs Rd in patients with previous exposure to bortezomib, a 3-month improvement in patients with disease refractory to bortezomib, a 15-month improvement in patients with previous thalidomide exposure, an 11-month improvement in patients refractory to thalidomide, a 5-month improvement in patients with previous lenalidomide exposure and a 2-month improvement in patients refractory to lenalidomide. Patients in the KRd group achieved a substantially higher CR or better rate than those in the Rd group, irrespective of the number of previous lines of therapy or the type of previous treatment. In the KRd group, the rate of CR or better was consistent across the number of previous lines of therapy and the type of previous treatment.

As expected, in both treatment groups, absolute median PFS and ORRs were observed to be generally better among patients who had received less previous treatment exposure. In the KRd group, lack of previous exposure to bortezomib, lack of previous exposure to lenalidomide and having one previous line therapy were associated with approximately 6-, 9- and 4-month longer median PFS durations compared with those in patients with previous exposure to bortezomib, previous exposure to lenalidomide and ⩾2 previous lines of therapy, respectively. In the Rd group, lack of previous exposure to bortezomib, lack of previous exposure to lenalidomide, and having one previous line therapy were associated with approximately 2-, 4- and 1-month longer median PFS durations compared with those in patients with previous exposure to bortezomib, previous exposure to lenalidomide and ⩾2 previous lines of therapy, respectively. These findings are consistent with a previous report on the clinical course of patients with relapsed MM, where it was observed that the event-free survival rate decreased with each additional line of therapy.^5^ It has been hypothesized that the decline in event-free survival rate with consecutive regimens reflects acquired drug resistance and the presence at relapse of tumor cells with an aggressive phenotype.^5, 12^ Resistance to the same agent or class of agent may have affected absolute efficacy outcomes in patients previously exposed to bortezomib or lenalidomide in the ASPIRE study. Although carfilzomib has been shown to be effective in patients failing bortezomib-based regimens and to be a more active proteasome inhibitor compared with bortezomib,^13, 14^ cross-resistance between these two proteasome inhibitors may exist.^12^ In the pivotal phase 2 study of single-agent carfilzomib in patients with relapsed and refractory MM, the ORR was lower in patients with ⩾2 vs <2 previous lines of bortezomib-based therapy (18.5% vs 29.5%).^15^

KRd reduced the risk of disease progression or death vs Rd across all subgroups analyzed. This benefit was most prominent in patients refractory to thalidomide (HR 0.599; 95% CI: 0.390, 0.918; *P*=0.0089) likely due to the relatively short median PFS observed in the Rd arm. The benefit of KRd vs Rd was least evident in patients with disease refractory to bortezomib (HR 0.799; 95% CI: 0.492, 1.297; *P*=0.1810), which is expected, given possible cross-resistance between bortezomib and carfilzomib. In addition, patients who were refractory to lenalidomide had only a 2-month improvement in median PFS with KRd vs Rd.

The safety profile of KRd vs Rd in patients with 1 and ⩾2 previous lines of therapy was consistent with those reported for the overall population,^7^ and no new or clinically significant safety signals were identified. In patients with 1 or⩾2 previous lines of therapy, cardiac and renal AEs were observed at rates consistent with those of previous carfilzomib studies.^6, 16^ Although patients with MM have been found to develop an increasing number of comorbidities and disease-related complications with additional lines of therapy,^3^ the safety profile of KRd in patients with 1 or ⩾2 previous lines of therapy was similar.

In addition to carfilzomib, there are many new approved agents for relapsed or refractory MM including ixazomib, elotuzumab and daratumumab. Thus, it will be important for future studies to understand how to best utilize these agents to optimize their benefits in treating patients with relapsed or refractory MM. Future trials evaluating combinations of these agents, including those combining daratumumab and carfilzomib, may also provide important information regarding optimal treatment strategies.

In conclusion, this subgroup analysis of the ASPIRE study indicated that KRd had a favorable benefit–risk profile compared with Rd, irrespective of previous treatment history. KRd should be considered an appropriate treatment option for patients in their first relapse, those who have ⩾2 previous lines of therapy, and those previously exposed to and/or refractory to bortezomib, thalidomide or lenalidomide.

## Figures and Tables

**Table 1:** Baseline demographic and clinical characteristics (ITT population)

*Characteristic*	*1 previous line of therapy*		⩾*2 previous lines of therapy*	
	*KRd* n=*184*	*Rd* n=*157*	*KRd* n=*212*	*Rd* n=*239*
Median age, years (range)	65.0 (40.0–87.0)	66.0 (40.0–91.0)	62.0 (38.0–87.0)	64.0 (31.0–87.0)
				
*Age cohort,* n *(%)*				
18–64 years	86 (46.7)	68 (43.3)	125 (59.0)	120 (50.2)
⩾ 65 years	98 (53.3)	89 (56.7)	87 (41.0)	119 (49.8)
				
*ECOG PS,* n *(%)*				
≤ 1	163 (88.6)	145 (92.4)	193 (91.0)	216 (90.4)
2	21 (11.4)	12 (7.6)	19 (9.0)	23 (9.6)
				
*Cytogenetic risk*^a^*,* n *(%)*				
High	23 (12.5)	18 (11.5)	25 (11.8)	34 (14.2)
Standard	70 (38.0)	72 (45.9)	77 (36.3)	98 (41.0)
Unknown	91 (49.5)	67 (42.7)	110 (51.9)	107 (44.8)
				
*Creatinine clearance*				
Mean±s.d., ml/min	83.0±26.2	80.6±27.7	86.7±31.1	89.4±31.3
Distribution, *n* (%)				
30 to <50 ml/min	8 (4.3)	21 (13.4)	17 (8.0)	10 (4.2)
⩾ 50 ml/min	175 (95.1)	134 (85.4)	195 (92.0)	224 (93.7)
Unknown/other	1 (0.5)	2 (1.3)	0	5 (2.1)
				
*Serum β*_*2*_*-microglobulin,* n *(%)*				
< 2.5 mg/l	35 (19.0)	24 (15.3)	42 (19.8)	53 (22.2)
⩾ 2.5 mg/l	149 (81.0)	133 (84.7)	170 (80.2)	186 (77.8)
				
*Previous therapy,* n *(%)*^b^				
Bortezomib	93 (50.5)	73 (46.5)	168 (79.2)	187 (78.2)
Refractory to bortezomib in any previous regimen^c^	20 (10.9)	6 (3.8)	40 (18.9)	52 (21.8)
Thalidomide	64 (34.8)	52 (33.1)	112 (52.8)	119 (49.8)
Refractory to thalidomide in any previous regimen^c^	10 (5.4)	15 (9.6)	51 (24.1)	50 (20.9)
Lenalidomide	34 (18.5)	21 (13.4)	45 (21.2)	57 (23.8)

Abbreviations: ECOG PS, Eastern Cooperative Oncology Group Performance Status; ITT, intent-to-treat; KRd, carfilzomib, lenalidomide and dexamethasone; Rd, lenalidomide and dexamethasone.

a The high-risk group consisted of patients with the genetic subtype t(4;14) or t(14;16) or with deletion 17p in 60% or more of plasma cells, according to central review of bone marrow samples obtained at study entry. The standard-risk group consisted of patients without t(4;14) or t(14;16) and with deletion 17p in <60% of plasma cells.

b Patients must not have progressed during the first 3 months of treatment with prior Rd or progressed at any time if Rd was the most recent line of prior treatment. If a patient progressed during any bortezomib-containing regimen, they were eligible to enroll if the progression date occurred after discontinuation of bortezomib.

c Refractory disease was defined as less than a minimal response to, or progression during therapy or within 60 days after completion of therapy.

**Table 2:** Responses by previous lines of therapy (1 vs ⩾2) (ITT population)

	*1 previous line of therapy*		⩾ *2 previous lines of therapy*	
	*KRd* n=*184*	*Rd* n=*157*	*KRd* n=*212*	*Rd* n=*239*
*Best overall response,* n *(%)*^a^				
sCR	23 (12.5)	5 (3.2)	33 (15.6)	12 (5.0)
CR	39 (21.2)	6 (3.8)	31 (14.6)	14 (5.9)
VGPR	78 (42.4)	57 (36.3)	73 (34.4)	66 (27.6)
PR	20 (10.9)	42 (26.8)	48 (22.6)	62 (25.9)
*ORR,* n *(%)*^a^	160 (87.0)	110 (70.1)	185 (87.3)	154 (64.4)
95% CI^b^	81.2–91.5%	62.2–77.1%	82.0–91.4%	58.0–70.5%
OR for KRd vs Rd (95% CI)	2.85 (1.65–4.93)		3.78 (2.33–6.13)	

Abbreviations: CI, confidence interval; CR, complete response; ITT, intent-to-treat; KRd, carfilzomib, lenalidomide and dexamethasone; OR, odds ratio; ORR, overall response rate; PR, partial response; Rd, lenalidomide and dexamethasone; sCR, stringent complete response; VGPR, very good partial response.

a Determined by independent review committee according to International Myeloma Working Group Uniform Response Criteria. Patients evaluated for ORR had a best overall response of PR or better.

b Clopper−Pearson interval.

**Table 3:** Response by type of previous treatment (ITT population)

	*Previous bortezomib*		*No previous bortezomib*	
	*KRd* n=*261*	*Rd* n=*260*	*KRd* n=*135*	*Rd* n=*136*
*Best overall response,* n *(%)*				
sCR	31 (11.9)	12 (4.6)	25 (18.5)	5 (3.7)
CR	47 (18.0)	13 (5.0)	23 (17.0)	7 (5.1)
VGPR	97 (37.2)	77 (29.6)	54 (40.0)	46 (33.8)
PR	50 (19.2)	63 (24.2)	18 (13.3)	41 (30.1)
*ORR,* n *(%)*^a^	225 (86.2)	165 (63.5)	120 (88.9)	99 (72.8)
95% CI^b^	81.4–90.1%	57.3–69.3%	82.3–93.6%	64.5–80.1%
OR for KRd vs Rd (95% CI)	3.60 (2.33–5.55)		2.99 (1.55–5.76)	

	*Previous thalidomide*		*No previous thalidomide*	
	*KRd* n=*176*	*Rd* n=*171*	*KRd* n=*220*	*Rd* n=*225*
*Best overall response,* n *(%)*				
sCR	32 (18.2)	8 (4.7)	24 (10.9)	9 (4.0)
CR	28 (15.9)	8 (4.7)	42 (19.1)	12 (5.3)
VGPR	63 (35.8)	41 (24.0)	88 (40.0)	82 (36.4)
PR	33 (18.8)	47 (27.5)	35 (15.9)	57 (25.3)
*ORR,* n *(%)*^a^	156 (88.6)	104 (60.8)	189 (85.9)	160 (71.1)
95% CI^b^	83.0−92.9%	53.1−68.2%	80.6−90.2%	64.7−76.9%
OR for KRd vs Rd (95% CI)	5.03 (2.88–8.78)		2.48 (1.54–3.99)	

	*Previous lenalidomide*		*No previous lenalidomide*	
	*KRd* n=*79*	*Rd* n=*78*	*KRd* n=*317*	*Rd* n=*318*
*Best overall response,* n *(%)*				
sCR	8 (10.1)	3 (3.8)	48 (15.1)	14 (4.4)
CR	11 (13.9)	1 (1.3)	59 (18.6)	19 (6.0)
VGPR	25 (31.6)	14 (17.9)	126 (39.7)	109 (34.3)
PR	20 (25.3)	21 (26.9)	48 (15.1)	83 (26.1)
*ORR,* n (%)^a^	64 (81.0)	39 (50.0)	281 (88.6)	225 (70.8)
95% CI^b^	70.6–89.0%	38.5–61.5%	84.6–91.9%	65.4–75.7%
OR for KRd vs Rd (95% CI)	4.27 (2.08–8.73)		3.23 (2.11–4.92)	

Abbreviations: CI, confidence interval; CR, complete response; ITT, intent-to-treat; KRd, carfilzomib, lenalidomide and dexamethasone; OR, odds ratio; ORR, overall response rate; PR, partial response; Rd, lenalidomide and dexamethasone; sCR, stringent complete response; VGPR, very good partial response.

a Determined by Independent Review Committee according to International Myeloma Working Group Uniform Response Criteria. Patients evaluated for ORR had a best overall response of PR or better.

b Clopper−Pearson interval.

**Table 4:** Adverse events of any grade by lines of therapy (safety population)

	*1 previous line of therapy*		⩾ *2 previous lines of therapy*	
	*KRd* n=*182*	*Rd* n=*154*	*KRd* n=*210*	*Rd* n=*235*
*AEs occurring in* ⩾*10% of patients in any subgroup (preferred terms),* n *(%)*				
Diarrhea	83 (45.6)	59 (38.3)	83 (39.5)	72 (30.6)
Anemia	82 (45.1)	66 (42.9)	85 (40.5)	89 (37.9)
Fatigue	65 (35.7)	45 (29.2)	64 (30.5)	74 (31.5)
Neutropenia	59 (32.4)	45 (29.2)	89 (42.4)	86 (36.6)
Muscle spasms	55 (30.2)	28 (18.2)	49 (23.3)	54 (23.0)
URTI	54 (29.7)	29 (18.8)	58 (27.6)	46 (19.6)
Cough	50 (27.5)	29 (18.8)	63 (30.0)	38 (16.2)
Pyrexia	49 (26.9)	33 (21.4)	63 (30.0)	48 (20.4)
Thrombocytopenia	49 (26.9)	32 (20.8)	65 (31.0)	56 (23.8)
Nasopharyngitis	48 (26.4)	28 (18.2)	36 (17.1)	35 (14.9)
Hypokalemia	45 (24.7)	25 (16.2)	63 (30.0)	27 (11.5)
Edema peripheral	45 (24.7)	28 (18.2)	40 (19.0)	47 (20.0)
Constipation	43 (23.6)	27 (17.5)	36 (17.1)	40 (17.0)
Dyspnea	42 (23.1)	22 (14.3)	34 (16.2)	36 (15.3)
Insomnia	38 (20.9)	23 (14.9)	37 (17.6)	40 (17.0)
Asthenia	35 (19.2)	29 (18.8)	35 (16.7)	27 (11.5)
Nausea	34 (18.7)	26 (16.9)	44 (21.0)	29 (12.3)
Hypocalcemia	33 (18.1)	16 (10.4)	30 (14.3)	29 (12.3)
Pneumonia	31 (17.0)	19 (12.3)	37 (17.6)	37 (15.7)
Bronchitis	29 (15.9)	21 (13.6)	45 (21.4)	33 (14.0)
Back pain	27 (14.8)	35 (22.7)	40 (19.0)	43 (18.3)
Hypophosphatemia	27 (14.8)	13 (8.4)	25 (11.9)	16 (6.8)
Hyperglycemia	26 (14.3)	17 (11.0)	23 (11.0)	21 (8.9)
Rash	25 (13.7)	23 (14.9)	27 (12.9)	37 (15.7)
Arthralgia	22 (12.1)	27 (17.5)	27 (12.9)	24 (10.2)
Dizziness	22 (12.1)	19 (12.3)	26 (12.4)	25 (10.6)
Pain in extremity	22 (12.1)	19 (12.3)	24 (11.4)	22 (9.4)
Headache	21 (11.5)	9 (5.8)	32 (15.2)	22 (9.4)
Hypertension	21 (11.5)	7 (4.5)	35 (16.7)	20 (8.5)
RTI	20 (11.0)	17 (11.0)	23 (11.0)	22 (9.4)
Vomiting	20 (11.0)	16 (10.4)	27 (12.9)	16 (6.8)
Decreased appetite	19 (10.4)	17 (11.0)	25 (11.9)	18 (7.7)
Bone pain	15 (8.2)	18 (11.7)	24 (11.4)	18 (7.7)
				
*AEs of interest (grouped terms),* n *(%)*				
Cardiac failure^a^	10 (5.5)	8 (5.2)	15 (7.1)	8 (3.4)
Ischemic heart disease^b^	12 (6.6)	7 (4.5)	11 (5.2)	11 (4.7)
Acute renal failure^c^	20 (11.0)	10 (6.5)	13 (6.2)	18 (7.7)

Abbreviations: AE, adverse event; CAD, coronary artery disease; KRd, carfilzomib, lenalidomide and dexamethasone; MI, myocardial infarction; Rd, lenalidomide and dexamethasone; RTI, respiratory tract infection; URTI, upper respiratory tract infection.

a Included: cardiac failure, congestive cardiac failure, pulmonary edema, hepatic congestion, cardiopulmonary failure, acute pulmonary edema, acute cardiac failure and right ventricular failure.

b Included: angina pectoris, MI, acute MI, increased blood creatinine phosphokinase, CAD, myocardial ischemia, coronary artery occlusion, increased troponin, increased troponin T, acute coronary syndrome, abnormal cardiac stress test, cardiomyopathy stress, unstable angina, coronary artery stenosis, abnormal electrocardiogram ST-T segment and abnormal electrocardiogram T wave.

c Included: acute renal failure, renal failure, renal impairment, azotemia, oliguria, anuria, toxic nephropathy and prerenal failure.

**Table 5:** Adverse events grade⩾3 by lines of therapy (safety population)

	*1 previous line of therapy*		⩾ *2 previous lines of therapy*	
	*KRd* n=*182*	*Rd* n=*154*	*KRd* n=*210*	*Rd* n=*235*
*Grade*⩾*3 AEs occurring in*⩾*3% of patients in any subgroup (preferred terms),* n *(%)*				
Neutropenia	48 (26.4)	34 (22.1)	68 (32.4)	69 (29.4)
Anemia	31 (17.0)	30 (19.5)	39 (18.6)	37 (15.7)
Thrombocytopenia	28 (15.4)	18 (11.7)	37 (17.6)	30 (12.8)
Pneumonia	22 (12.1)	16 (10.4)	27 (12.9)	25 (10.6)
Hypophosphatemia	18 (9.9)	8 (5.2)	15 (7.1)	10 (4.3)
Hypokalemia	14 (7.7)	11 (7.1)	23 (11.0)	8 (3.4)
Fatigue	13 (7.1)	10 (6.5)	17 (8.1)	15 (6.4)
Hyperglycemia	11 (6.0)	10 (6.5)	9 (4.3)	8 (3.4)
Cataract	10 (5.5)	4 (2.6)	5 (2.4)	6 (2.6)
Pulmonary embolism	8 (4.4)	4 (2.6)	4 (1.9)	5 (2.1)
RTI	8 (4.4)	3 (1.9)	8 (3.8)	5 (2.1)
Asthenia	7 (3.8)	5 (3.2)	7 (3.3)	3 (1.3)
Hypertension	7 (3.8)	1 (0.6)	10 (4.8)	6 (2.6)
Hypocalcemia	7 (3.8)	3 (1.9)	6 (2.9)	3 (1.3)
Insomnia	7 (3.8)	4 (2.6)	4 (1.9)	7 (3.0)
Diarrhea	6 (3.3)	6 (3.9)	9 (4.3)	10 (4.3)
Leukopenia	6 (3.3)	5 (3.2)	6 (2.9)	11 (4.7)
Lymphopenia	6 (3.3)	3 (1.9)	5 (2.4)	5 (2.1)
Decreased platelet count	6 (3.3)	3 (1.9)	6 (2.9)	6 (2.6)
Rash	4 (2.2)	5 (3.2)	1 (0.5)	1 (0.4)
Decreased neutrophil count	4 (2.2)	1 (0.6)	8 (3.8)	10 (4.3)
Dyspnea	4 (2.2)	4 (2.6)	7 (3.3)	3 (1.3)
Syncope	3 (1.6)	2 (1.3)	2 (1.0)	8 (3.4)
				
*AEs of interest (grouped terms),* n *(%)*				
Cardiac failure^a^	6 (3.3)	3 (1.9)	9 (4.3)	4 (1.7)
Ischemic heart disease^b^	9 (4.9)	2 (1.3)	4 (1.9)	6 (2.6)
Acute renal failure^c^	6 (3.3)	5 (3.2)	7 (3.3)	7 (3.0)

Abbreviations: AE, adverse event; KRd, carfilzomib, lenalidomide and dexamethasone; MI, myocardial infarction; Rd, lenalidomide and dexamethasone; RTI, respiratory tract infection.

a Included: cardiac failure, congestive cardiac failure, pulmonary edema, hepatic congestion, cardiopulmonary failure, acute pulmonary edema, acute cardiac failure and right ventricular failure.

b Included: angina pectoris, MI, acute MI, coronary artery occlusion, acute coronary syndrome, abnormal cardiac stress test, cardiomyopathy stress, unstable angina and coronary artery stenosis.

c Included: acute renal failure, renal failure, renal impairment and azotemia.
